# Tunisian *Artemisia campestris* L.: a potential therapeutic agent against myeloma - phytochemical and pharmacological insights

**DOI:** 10.1186/s13007-024-01185-4

**Published:** 2024-05-02

**Authors:** Inès Limam, Ridha Ghali, Mohamed Abdelkarim, Anis Ouni, Manel Araoud, Mouaadh Abdelkarim, Abderrazek Hedhili, Fatma Ben-Aissa Fennira

**Affiliations:** 1 PRF of Onco-Hematology, Faculty of medicine of Tunis, Tunis El Manar University, Tunis, Tunisia; 2 Human genetics laboratory, Faculty of medicine of Tunis, Tunis El Manar University, Tunis, Tunisia; 3 Research Laboratory of Toxicology and Environment, CAMU of Tunis, Tunis, LR12SP07 Tunisia; 4 Higher institute of Biotechnology of Sidi Thabet, Manouba University, Manouba, Tunisia; 5 College of General Education, University of Doha for Science & Technology, PO Box 24449, Doha, Qatar

**Keywords:** *Artemisia campestris*, Polyphenol, Phytosterol, Terpene, Antioxidant, Anticancer, Multiple myeloma, Antibacterial, Antifungal.

## Abstract

**Background:**

*Artemisia campestris* L. (AC) leaves are widely recognized for their importance in traditional medicine. Despite the considerable amount of research conducted on this plant overworld, the chemical composition and the biological activity of the leaves grown in Tunisia remains poorly investigated. In this study of AC, a successive extraction method was employed (hexane, ethyl acetate and methanol) to investigate its bioactive constituents by LC-MS analysis, and their antioxidant, antibacterial, antifungal, and anticancer activities.

**Results:**

Data analysis revealed diverse compound profiles in AC extracts. Methanolic and ethyl acetate extracts exhibited higher polyphenolic content and antioxidant activities, while Hexane showed superior phytosterol extraction. Ethyl acetate extract displayed potent antibacterial activity against multi-resistant *Staphylococcus aureus* and *Pseudomonas aeruginosa*. Additionally, all extracts demonstrated, for the first time, robust antifungal efficacy against *Aspergillus flavus* and *Aspergillus niger*. Cytotoxicity assays revealed the significant impact of methanolic and ethyl acetate extracts on metastatic breast cancer and multiple myeloma, examined for the first time in our study. Moreover, further analysis on multiple myeloma cells highlighted that the ethyl acetate extract induced apoptotic and necrotic cell death and resulted in an S phase cell cycle blockage, underscoring its therapeutic potential.

**Conclusions:**

This investigation uncovers novel findings in Tunisian AC, notably the identification of lupeol, oleanolic acid, ursolic acid, stigmasterol and β-sitosterol. The study sheds light on the promising role of AC extracts in therapeutic interventions and underscores the need for continued research to harness its full potential in medicine and pharmaceutical development.

**Supplementary Information:**

The online version contains supplementary material available at 10.1186/s13007-024-01185-4.

## Background

Cancer remains one of the most significant health challenges in the 21st century, affecting diverse populations across various social, ethnic, and economic backgrounds. Despite advances in early detection and awareness, cancer still ranks as the second leading cause of death worldwide, following cardiovascular diseases [1]. The tumor cells possess replicative immortality mechanisms [2] and exhibit increased resistance to cell death through the regulation of anti and pro-apoptotic proteins [3]. Breast cancer stands as a major cause of cancer-related deaths among women globally. Approximately 15–20% of breast cancer cases fall under the triple-negative breast cancer subtype (TNBC), known for its aggressiveness and a higher rates of relapse [4]. On another front, multiple myeloma, a disease of terminally differentiated plasma cells, ranks as the second most common hematological malignancy after non-Hodgkin lymphoma [5]. For patients with this cancer, treatment outcomes still remain suboptimal, as many develop resistance and succumb to the disease [6]. These challenges underscore the urgency of exploring new therapeutic strategies to overcome drug resistance [7]. In addition to drug resistance, research on the involvement of infections and bacteria in cancer has gained considerable momentum in recent years. Certain bacterial infections have been identified as potential contributors to the development of specific types of cancer, holding significant implications for cancer prevention and treatment [8].

Natural molecules, especially those derived from plants, have garnered significant interest in the treatment of cancer, infections, and bacterial-related ailments [9–11]. Plant-based compounds possess a diverse array of bioactive constituents that exhibit potential therapeutic effects. In cancer treatment, these natural molecules have shown promise as anticancer agents, either by inducing apoptosis in cancer cells, inhibiting tumor growth, or interfering with specific signaling pathways [12, 13]. Moreover, the use of plant-derived compounds as adjuvants in chemotherapy and radiation therapy has been explored to enhance treatment efficacy and reduce adverse effects [14]. In the context of infections and bacteria, natural molecules have demonstrated remarkable antimicrobial properties. Plant extracts and essential oils have been found to possess potent antibacterial and antifungal activities, making them valuable alternatives or complements to conventional antibiotics [15, 16]. The rich reservoir of bioactive compounds in plants offers a promising avenue for the development of novel therapeutics, emphasizing the importance of further research to harness the full potential of these natural molecules in combating cancer and infectious diseases.

Members of the *Artemisia* genus are widely recognized for their importance in traditional medicine. This enduring herb, belonging to the Asteraceae family, is distributed worldwide [17]. *Artemisia campestris* L. (AC), a polymorphic species comprising various subspecies and varieties, possesses subtle aromatic characteristics [18]. This species is found in the north of Africa, and particularly in the middle and the south of Tunisia where it is commonly referred to as “T’gouft” [18, 19]. The leaves of this plant, generally collected in summer months (July/August), are widely used in traditional medicine due to their noted digestive, analgesic, and antihypertensive attributes [19]. As evidenced by the existing literature, numerous studies have underscored the antivenin, anti-inflammatory, antirheumatic, and antimicrobial properties of AC species [20]. Notably, a particular emphasis has been placed on its efficacy in treating ailments of the digestive system, especially gastroesophageal disorders [21].

In the realm of traditional medicine, plant extracts are commonly prepared through infusion techniques [22]. Many studies on AC have employed aqueous and alcohol extraction methods or have focused on essential oils [18]. These extraction processes yield a range of compounds from AC, including flavonoids, chromones, and acetophenones, which contribute to the plant’s diverse biological properties [18]. Notably, recent research by Cheraif et al. underscored the anti-cancer potential of AC essential oil [23]. It is well-established that plant extractions vary depending on the solvent employed, producing distinct phytomolecules based on the solvent’s polarity [24]. Building on this knowledge and recognizing the plant’s multifaceted uses, our study sought to explore the biological activities of AC leaves, when extracted using solvents of increasing polarity, namely hexane (Hex), ethyl acetate (EtAC) and methanol (MeOH). Our investigation encompassed an examination of the plant’s antibacterial, antioxidant, and antifungal properties. Additionally, we delved into its potential anticancer effects against breast and myeloma cells, in conjunction with an analysis of its overarching chemical composition.

## Materials and methods

### Sample collection

The aerial parts (stems and leaves) of *Artemisia campestris* L were collected in July 2020 from the Foussana area in west-central Tunisia (Kasserine Governorate) and air-dried in the shade. The species were identified for this scientific study by using the Tunisia flora [25–27]. Samples were deposited at the herbarium of the Research Laboratory of Toxicology and Environment to assess a voucher number. The leaves were then separated from stems, collected, crushed, and subsequently stored in hermetically sealed plastic bags at -18 °C until analysis.

### Biological materiel’s

To examine the antifungal properties, two strains were used: an *Aspergillus flavus* strain (SRRC 1000 A) and *Aspergillus niger* strain (CBS 513), both of which had been previously isolated from wheat samples and were stored in the Toxicology Laboratory at Tunis. Additionally, the strains of *Pseudomonas aeruginosa* and *Staphylococcus aureus* were purchased from American Type Culture Collection (ATCC) (serial number of 27,853 and 29,213, respectively) and employed to investigate the antibacterial properties of AC extracts. The bacterial strains were cultivated on TBS (trypticase soy broth) medium. For antitumor activities, myeloma cell lines consisted of U266, a model of multiple myeloma derived from peripheral blood of a 53-year-old man with refractory, and JJN3, established from the bone marrow of a 57-year-old woman with plasma cell leukemia related to multiple myeloma, were graciously provided by Professor Brigitte Sola [28]. MDA-MB-231, a human breast cancer epithelial cell line established from a pleural effusion of a 51-year-old woman with metastatic adenocarcinoma was purchased from ATCC (ATCC HTB-26) [4]. U266 and JJN3 cell lines were maintained in standard culture condition using RPMI 1640 medium while MDA-MB-231 cells were cultured in DMEM medium.

### Chemicals

HPLC-grade solvents and chemicals such as dimethyl sulfoxide (DMSO), 2,2-diphenyl-1-picrylhydrazyl (DPPH), formic acid, 3-(4,5-dimethylthiazol-2-yl)-2,5-diphenyltetrazolium bromide (MTT), propidium iodide (PI), 4’,6-diamidino-2-phénylindole (DAPI) and acridine orange were purchased from Sigma-Aldrich (St. Louis, MO, USA). Dehydrated microbiology media were purchased from Scharlau (Barcelona, Spain). Analytical standard solutions of phenolic acids, flavonoids, terpenes and phytosterols as presented in Table 1 were procured from Sigma Chemical Co. (St. Louis, MO, USA). Cell culture reagents, including RPMI 1640 and DMEM mediums, fetal bovine serum (FBS), penicillin 0.1 µg/mL and streptomycin 0.1 µg/µL, phosphate buffered-saline (PBS), and DMSO were ordered from PAN-Biotech (Aidenbach, Germany). Purified water was obtained using a MultiQ system from Millipore, (Darmstadt, Allemagne).

**Table 1:** The different compounds explored in various AC extracts

Phenolic acids	Flavonoids	Terpenes
Caffeic acid Chlorogenic acid Ferulic Acid Gallic acid Protocatechuic acid Quinic acid Rosmarinic acid Salvianolic acid A Syringic acid Trans-Cinnamic acid 1,3-Dicaffeoylquinic acid 3,4-Dicaffeoylquinic acid 4,5-Dicaffeoylquinic acid	Acacetin Apigenin Apigenin-7-O-glucoside Catechin Cirsilineol Cirsiliol Epicathechin Luteolin Luteolin-7-O-glucoside Quercetin Rutin Silymarin	Lupeol Oleanolic acid Ursolic acid
		**Phytosterols**
		Stigmasterol β-sitosterol

### Extracts preparation

The crude extract was obtained through a process of serial exhaustive extraction, which entails a series of successive extractions of the plant material using three solvents with increasing polarity. This methodology aims to retrieve biomolecules from the plant material effectively as previously described [29]. For this procedure, 25 g of finely ground leaves material, placed in separate Erlenmeyer flasks, were subjected to maceration in 200 mL of Hex under shaking conditions for a duration of 24 h. The maceration was carried out, with agitation occurring at 200 rpm using an I’ROLL shaking rotator from Accumax Lab Devices Pvt. Ltd. Following the maceration process, the supernatant was filtered using filter paper. The solid residues that remained after filtration were then subjected to gradual extraction using EtAC firstly and then MeOH. Upon each extraction, the obtained supernatant was collected and subsequently dried at 40 °C using a rotary evaporator from Heidolph Instruments GmbH. The extractions were carried out in triplicate, and the yields of dry extract residues (DE) are expressed as mean ± standard deviation using the following equation: Yield % = (DE mass / Plant material initial mass) * 100.

The resulting DE were carefully collected, their masses were recorded, and they were subsequently stored at a temperature of 4 °C. The different obtained DE were reconstituted in 2% DMSO (for the assessment of biological activities) or in MeOH (for chromatographic and anticancer analyses) to achieve mother concentrations of 0.25 mg/mL.

### LC-MS analysis

Quantitative analysis of various target components in different fractions of AC was performed using a Shimadzu 8020 UPLC-ESI-MS system (Shimadzu, Kyoto, Japan). This system was equipped with a binary pump Nexera XR LC-20AD. The entire system, including the control of LC and MS hardware, as well as the processing of chromatographic and spectral data, was managed using the Shimadzu LabSolutions LC-MS software (Shimadzu, Kyoto, Japan). Accurate identification was achieved by matching mass spectra with those obtained from standard solutions, thereby ensuring precise and reliable result (supplementary data S1).

#### Polyphenols quantification

Quantitative determination of thirteen phenolic acids and twelve flavonoids was conducted on extracts using a previously validated methods [30]. Chromatographic separation was carried out on a reverse-phase C18 column (100 × 2.1 mm x 3 μm) maintained at a constant temperature of 40 °C. The mobile phase consisted of a mixture of aqueous solutions: 0.1% formic acid (A) and a solution of 0.1% formic acid in MeOH (B). The separation was achieved through a linear gradient elution, at a constant flow rate of 0.4 mL/min, starting from 10% B and gradually increasing to 100% B over a period of 45 min, followed by a constant 100% B from 45 to 50 min and from 100% B and to 10% B over a period of 5 min.

Mass spectra were acquired in positive and negative ion modes within a mass-to-charge ratio (m/z) range of 50 to 1200. Nitrogen was utilized as the nebulizing gas at a flow rate of 1.2 L/min. The drying line temperature was maintained at 250 °C. The capillary voltage was set at -3.5 V, while the detector voltage was set at 1.1 V.

#### Phytosterols and terpenes quantification

Phytosterols and terpenes were separated using a LC-MS system equipped with an AQUASIL C18 column (150 mm × 3 mm x 2 μm) at a flow rate of 0.5 mL/min. The mobile phase consisted of a mixture of acetic acid and water (90/10: v/v) as solvent A and 10% MeOH-water as solvent B. For the elution of phytosterols, the process involved a linear gradient starting from 90 to 95% A over 20 min. Subsequently, it moved from 95 to 100% A over 2 min, maintaining a constant 100% B phase for 8 min, before reverting to the initial conditions for a final 5 min. In the case of terpene separation, the elution gradient commenced with a linear transition from 75 to 87% B over 15 min, followed by a gradient shift from 87 to 100% B and a one-min hold at 100% B, before returning to the initial conditions with 75% B for 6 min.

For both methods, 5 µL injection volume was employed for each sample, and the column was maintained at 40 ºC. The MS detector operating method was the same for both molecules’ groups. The mass scans were performed by a negative electrospray ionization in selected ion monitoring (SIM) mode over the range from 50 to 1200 m/z with a source temperature of 400 °C, collision energy set at 35 ± 15 eV in positive mode, an ion spray voltage of 5.5 kV, ion source gas pressures of 50 psi, and collision gas pressure of 20 psi.

### Antioxidant properties

The DPPH free radical reduction method, as detailed by Kirby and Schmidt, was used [31]. This method allowed for the quantification of the extracts’ ability to scavenge free radicals and provided valuable insights into their potential antioxidant properties. In this procedure, 500 µl of each extract solution at 50 µg/mL was added to 375 µL of MeOH 99% and 125 µL of DPPH solution (0.2 mM). After shaking, the tubes were incubated at room temperature in dark for 60 min. After this incubation period, the absorbance at 517 nm was measured using T60 spectrophotometer (PG Instruments, Wibtoft, Leicestershire, UK). For comparative purposes, negative control tubes were also prepared. These control tubes contained only the DPPH solution without the addition of any extract. The entire test was conducted in triplicate to ensure accuracy and reliability. The results of the analysis were expressed as the percentage of DPPH radical inhibition.

### Antibacterial activity

The antibacterial efficacy of the extracts was assessed using the agar well diffusion method outlined by Celiktas et al. in 2007 [32]. Initially, the *Staphylococcus aureus* and *Pseudomonas aeruginosa* strains were cultivated in appropriate condition and allowed to grow. Then, the medium was subjected to centrifugation to recover the bacterial pellet which was diluted with PBS to achieve a concentration of 0.5–4.10^6^ CFU/mL. This solution was used for inoculating 10 mL of culture medium in a freshly prepared petri dish maintained at a temperature of 42 °C, and which was earlier cooled at 4 °C for a duration of 10 min, allowing the culture medium layer to solidify. Next, sixteen wells were superficially perforated at the surface of this solidified layer. In each of these wells, 5 µL of the different solvent extract solution, dissolved in isoamyl alcohol 30% at a concentration of 25 µg/mL, was deposited. Following this, the Petri dish was incubated for 3 h at 37 °C. The petri dish surface was subsequently overlaid with an additional 10 mL of the same medium, and the dish was once again cooled at 4 °C for 15 min to solidify the upper layer. The dish was then incubated for a further 18 h at 37 °C. The antibacterial activity was assessed based on the presence of bacterial growth inhibition zones surrounding the wells where the extracts had been placed. As a negative control, inhibition zones were determined for a sterile 30% isoamyl alcohol solution. To ensure robustness, all tests were conducted in triplicate.

### Antifungal activity

To assess antifungal effects, the solid agar diffusion method was employed [33]. In this procedure, 1 mL of the test extract (a series of dilutions were prepared of each extract) was combined with 19 mL of Dichloran Rose Bengal Chloramphenicol (DRBC) medium, and the resulting mixture was poured into petri dishes (with final concentration of 12.5, 25 and 50 µg/mL). In the center of each dish, a mycelial disc of approximately 0.6 cm in diameter, derived from a 5-day pre-culture of fungal strains, was aseptically positioned. Subsequently, the petri dishes were incubated at a temperature of 25 °C for a duration of 5 days. To provide context, control dishes were prepared. They consisted of culture medium, DMSO and mycelial discs, serving to evaluate medium fertility and estimate the growth rate of fungal strains without the inhibitory effects of extracts. Following the incubation period, the diameters of the mycelial growth on the various dishes were measured, and the percentage of growth inhibition was calculated using the following formula: mycelial growth inhibition (%) = 100 *(mycelial diameter growth (control) - mycelial diameter growth with the test extract)/ mycelial diameter growth in control.

### Cytotoxic activity

The cytotoxic potential of AC leaf extracts against MDA-MB-231, JJN3 and U266 cells was evaluated in vitro using MTT assay following established protocol [34]. The MTT assay was conducted to assess cytotoxicity in a dose- and time-dependent manner. Briefly, cells were seeded into 96-well plates and exposed to escalating concentrations (15.56, 31.125, 62.25, 125 and 250 µg/mL) of different solvent extracts for 24–48 h. Corresponding concentrations of DMSO were added to control wells. The MTT reagent was converted into formazan by active mitochondrial enzymes in viable cells. The absorbance (A) of the formazan was measured at 490 nm using a microplate reader (Model EXL800; BioTek, USA). The formula utilized to calculate cell viability was: % Inhibition = 100 - ((Absorbance of treated sample/Absorbance of untreated sample) * 100).

### Determination of cell death impact

The investigation into the impact of the EtAC extract on myeloma cells involved a comprehensive analysis of its influence on cell death, morphological nuclear changes, and cell cycle progression. Initially, cancer cells were exposed to 62.25–125 µg/mL of the EtAC extract for a 24-h culture period. Subsequent evaluation the type of cell death induced by the extracts was pursued using acridine orange/ PI double staining. This dual staining method allowed for the differentiation between apoptotic and necrotic cells, providing insights into the specific mode of cell death induced by the plant extract treatment. Following this, the determination of nuclear changes indicative of alterations in myeloma cells was conducted after DAPI staining and fluorescence microscopy analysis, following the methodology outlined by Limam and colleagues [13]. Lastly, the identification of changes in cell cycle phases through PI staining and flow cytometry exploration was performed as previously described procedures [28].

### Statistical analysis

Three independent analyses were performed for each experiment to ensure robustness and reliability. The acquired data were subjected to statistical processing using SPSS version 20 and GraphPad prism 5 softwares. During this analysis, mean values, and standard deviations (SD) were calculated. Statistical significance was determined based on a probability threshold of *p* < 0.05. This approach allowed for accurate evaluation and interpretation of the experimental results.

## Results

### Extraction yields

The extracts were derived through a series of sequential extractions, and subsequently, the yields of DE were calculated. The results revealed that EtAC and MeOH extracts displayed the most substantial yields, with respective values of 13.62 ± 4.92% and 10.74 ± 4.31%. In contrast, the Hex extract exhibited a notably lower yield of 2.60 ± 3.84%. These finding suggest that the extraction method using solvents like MeOH or EtAC is the most suitable for achieving both a higher yield and for recovering compounds with different polarities from the plant material. This conclusion is consistent with common practices in extraction methods, where the choice of solvent can significantly impact the efficiency of extraction and the range of compounds obtained.

### Phenolic acids content

The LC-MS analysis of thirteen phenolic acids in various extracts yielded results that are summarized in Table 2. Data revealed that each of the solvents used in the extraction process possesses a unique capability to extract specific molecules. MeOH emerged as the most effective solvent for the extraction of the majority phenolic acids. Its effectiveness was evidenced by its ability to recover ten out of the thirteen analyzed molecules, resulting in a cumulative concentration of 46.07 mg/g DE. This notable efficiency of MeOH can be attributed to its high polarity, which facilitates the extraction process. On the other hand, EtAC also proved competence in extracting a significant portion (6 out of 13) of phenolic acids, at lower concentrations. In contrast, Hex was unsuitable for the extraction of these molecules, as it only allowed for the recovery of a limited subset, which included rosmarinic, 1,3-dicaffeoylquinic, and quinic acids exclusively. Remarkably, with the MeOH extract, the most concentrated phenolic acids are identified as 4,5-dicaffeoylquinic and 3,4-dicaffeoylquinic acids with concentrations of 23.26 mg/g DE and 15.24 mg/g DE respectively. Interestingly, the maximum concentration of ferulic acid was obtained through extraction using EtAC, with a concentration of 0.16 mg/g DE (compared to 0.14 mg/g, with MeOH as the solvent).

**Table 2:** Results of phenolic acid quantification (mg/g of DE) of different fractions obtained from AC.

	Phenolic acid content (mg/g DE)		
Phenolic acids	Hex extract	EtAC extract	MeOH extract
Caffeic acid	ND	0.196 ± 0.02	0.25 ± 0.02
Chlorogenic acid	ND	0.038 ± 0.01	0.99 ± 0.08
Frulic acid	ND	0.16 ± 0.01	0.14 ± 0.01
Gallic acid	ND	ND	ND
Quinic acid	0.0406 ± 0.004	ND	5.49 ± 0.49
Protocatechuic acid	ND	0.12 ± 0.04	0.21 ± 0.02
Rosmarinic acid	0.02 ± 0.003	ND	0.03 ± 0.01
Syringic acid	ND	0.11 ± 0.01	0.44 ± 0.04
Salvionolic acid A	ND	0.01 ± 0.002	ND
Trans-Cinnamic acid	ND	ND	ND
1,3-Dicaffeoylquinic acid	0.03 ± 0.004	ND	0.02 ± 0.003
3,4-Dicaffeoylquinic acid	ND	ND	15.24 ± 1.37
4,5-Dicaffeoylquinic acid	ND	ND	23.26 ± 2.56

ND : Not detected

Each value is the average of three analyses ± SD

### Flavonoid contents

The analysis of flavonoids in the diverse extracts of AC were succinctly summarized in Table 3. When comparing different solvents for the extraction of these molecules, MeOH demonstrated proficiency in extracting most of the assessed molecules, recovering six out of the twelve, resulting in a cumulative concentration of 783.46 mg/g DE. Similarly, EtAC has proven to be effective in extracting the tested flavonoids, yielding a total concentration of 773.68 mg/g DE. In contrast, extracts obtained with Hex show a considerably lower total flavonoid concentration of 0.81 mg/g DE. Among the array of investigated flavonoids, noteworthy concentrations were observed for cirsiliol, cirsilineol, and luteolin, with respective concentrations of 746.03 mg/g DE, 27.67 mg/g DE, and 8.41 mg/g DE in the MeOH extract, and 743.213 mg/g DE, 22.69 mg/g DE, and 7.51 mg/g DE in the EtAC extract. Interestingly, quercetin was only poorly detected in the Hex extract, with a concentration of 0.033 mg/g DE.

**Table 3:** Total flavonoid contents (mg/g DE) in the various AC extracts

	Flavonoid concentrations (mg/g DE)		
Flavonoids	Hex extract	EtAC extract	MeOH extract
Acacetin	0.088 ± 0.01	0.26 ± 0.02	0.26 ± 0.02
Apigenin	ND	ND	ND
Apigenin-7-O-glucoside	ND	ND	ND
Catechin	ND	ND	ND
Cirsilineol	0.326 ± 0.03	22.69 ± 1.82	27.67 ± 2.21
Cirsiliol	ND	743.21 ± 59.46	746.03 ± 59.68
Epicathechin	ND	ND	ND
Quercetin-3-O-galactoside	0.033 ± 0.004	ND	ND
Luteolin	0.369 ± 0.03	7.51 ± 0,60	8.41 ± 0.67
Luteolin-7-O-glucoside	ND	ND	0.92 ± 0.07
Rutin	ND	ND	ND
Silymarin	ND	0.01 ± 0.003	0.14 ± 0.01

ND: Not detected

Values in columns are the average of three analyses ± SD

### Terpene contents

The quantification of three terpenes, namely lupeol, oleanolic, and ursolic acids, was conducted within the examined AC extracts, confirming their presence in this plant. The specific determined contents are presented in Table 4. Quantitative analysis revealed the highest terpene contents in the EtAC extract (40.41 mg/g DE), while the lowest contents were found in the MeOH extract (0.09 mg/g DE). Significantly, lupeol was only detected in the EtAC extract, with a concentration of 34.03 mg/g DE. Moreover, all three plant fractions contained oleanolic acid, with the highest contents recorded in the EtAC extract (6.37 mg/g DE), while ursolic acid was only poorly detected in Hex and MeOH extracts.

**Table 4:** Terpene contents in various AC extracts (mg/g DE)

Terpenes	Hex extract	EtAC extract	MeOH extract
Lupeol	0.00	34.03 ± 4.83	0.00
Oleanolic acid	3.57 ± 0.98	6.37 ± 1.46	0.06 ± 0.01
Ursolic acid	1.18 ± 0.24	0.00	0.03 ± 0.004

Values are mean ± SD of samples analyzed

### Phytosterol contents

The quantification of stigmasterol and β-sitosterol in the different solvent extracts has revealed their substantial presence in the studied plant. The significant abundance of these sterols in the lipid fraction explains their pronounced extraction by Hex (67.55 mg/g DE) and suggests a greater affinity to EtAC compared to MeOH (2.25 vs. 0.61 mg/g DE). The following table delineates their concentrations in the respective extracts (Table 5).

**Table 5:** Phytosterol levels in the three AC extracts (mg/g DE).

Extract	Stigmasterol (mg/g DE)	β-Sitosterol (mg/g DE)
**Hex**	53.75 ± 5.34	13.80 ± 2.23
**EtAC**	1.74 ± 0.37	0.51 ± 0.08
**MeOH**	0.44 ± 0.08	0.17 ± 0.09

Values are the average of three analyses ± SD

### Antiradical activity

The DPPH free radical reduction method was employed to quantify of the extracts’ capacity to neutralize free radicals, offering valuable insights into their potential antioxidant properties. All tested extracts of AC demonstrated remarkable anti-radical properties at 50 µg/mL. The MeOH extract exhibited the highest level of activity, recording an impressive 89.05 ± 1.26% radical scavenging (*p* < 0.05). The EtAC extract displayed a slightly lower activity, registering at 74.19 ± 0.59%. In contrast, the Hex extract exhibited the weakest anti-radical activity, with a value of 37.87 ± 0.83%.

#### Antibacterial activity

The antibacterial activities of AC extracts were assessed against multi-resistant reference strains of *Staphylococcus aureus* and *Pseudomonas aeruginosa* at a concentration of 25 µg/mL. These activities were observed by measuring the formation of inhibition zones around the wells containing the extracts. After an 18-h incubation period at 37 °C, the inhibition diameters were measured and ranged from 6 mm to 12 mm, as detailed in Table 6. Results demonstrated a strong inhibitory effect of the EtAC extract against both strains. *Staphylococcus aureus* proved to be the most sensitive bacteria strain, with a large zone of inhibition measuring 12.22 mm, while *Pseudomonas aeruginosa* exhibited an inhibition zone of 7.66 mm. Furthermore, the data also indicated the performance of Hex extract against the two multi-resistant strains, resulting in an inhibition zone of approximately 6.1 mm. In contrast, the MeOH extract did not exhibit any antibacterial activity against these reference strains. These findings highlight the potential of AC extracts, particularly the EtAC extract, as antibacterial agents, especially against challenging multi-resistant bacterial strains.

**Table 6:** Inhibition zone diameters (mm) induced by the three AC extracts against two bacterial strains

Extract	Tested Strains	Inhibition zone (mm)
**Hex**	*Staphylococcus aureus*	6.03 ± 1.42
	*Pseudomonas aeruginosa*	6.20 ± 2.21
**EtAC**	*Staphylococcus aureus*	12.22 ± 3.54
	*Pseudomonas aeruginosa*	7.66 ± 2.83
**MeOH**	*Staphylococcus aureus*	0.00
	*Pseudomonas aeruginosa*	0.00

Data presented as means ± SD of three individually analyses

### Antifungal activity

The solid agar diffusion method was employed to evaluate the impact of the investigated extracts on mycelial growth by calculating the percentage of inhibition, as described in the method section. The results of this analysis are presented in Table 7. Interestingly, a potent antifungal efficacy was observed with all three AC extracts against the tested fungal species. Notably, the *Aspergillus flavus* isolate displayed greater sensitivity to a concentration of 50 µg/mL of the different extracts, showing significantly higher inhibition values of 92.39%, 85,61% and 78.33% for the MeOH, EtAC and Hex extracts, respectively. Conversely, *Aspergillus niger* demonstrated reduced sensitivity, with maximum inhibition rates of 65.4%, 58.13% and 45.93% for the same extracts, respectively. Moreover, for the MeOH extract, even the lowest concentration used (12.5 µg/mL) maintained inhibitory effects of 73% and 58.14% on *Aspergillus flavus* and *Aspergillus niger*, respectively, indicating its significant antifungal potential. The other two extracts appear to exhibit dose-dependent effects. These results collectively underscore the potential of AC extracts as sources of effective antifungal agents, with their potency being influenced by both the choice of solvent and extract concentration.

**Table 7:** Percentage of growth inhibition of tested fungi induced by the different extract solutions

Extract concentration (µg/mL)		Tested fungi	
		Aspergillus flavus	Aspergillus niger
		Growth inhibition (%)	
Hex extract	50	78.33 ± 2.32	45.93 ± 3.92
	25	42.45 ± 2.82	22.15 ± 1.13
	12.5	39.8 ± 1.96	16.87 ± 1.12
EtAC extract	50	81.83 ± 2.54	58.13 ± 1.9
	25	65.64 ± 3.08	50.00 ± 2.08
	12.5	47.56 ± 2.9	11.42 ± 1.92
MeOH extract	50	92.39 ± 3.62	65.4 ± 2.52
	25	76.69 ± 2.22	60.36 ± 3.12
	12.5	73 ± 2.14	58.14 ± 1.87

Data in columns presented the average ± SD of three different experiments

### Cytotoxic activity

**Fig. 1:**
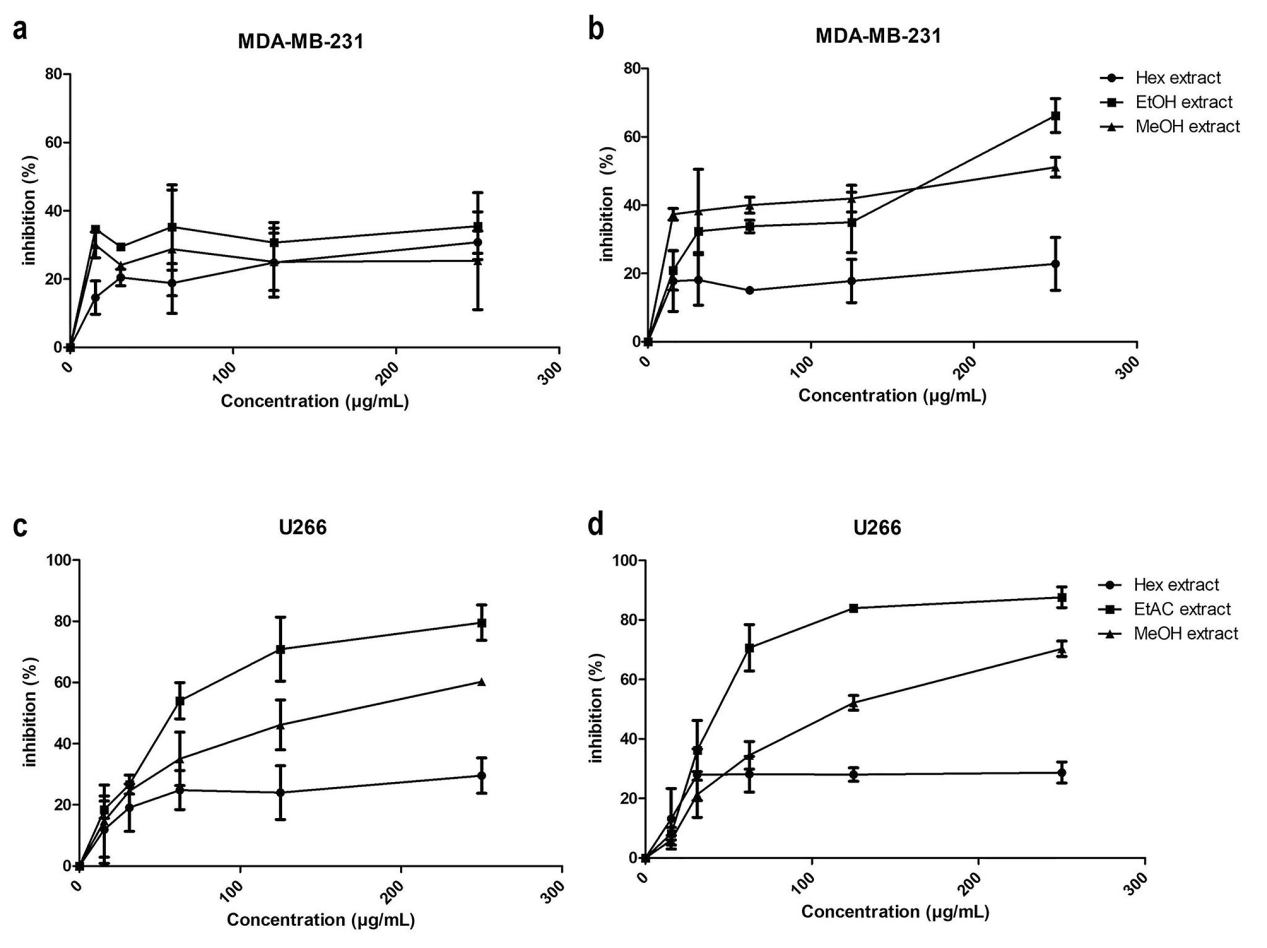
Cytotoxic potential of AC leaf extracts. The MTT assay was conducted to assess cytotoxicity in vitro against MDA-MB-231 (**a**, **b**) and U266 (**c**, **d**) cells. The dose response curves of inhibition of proliferation after 24 h (**a**, **c**) and 48 h (**b**, **d**) of individual treatments by escalating concentrations (15.56, 31.125, 62.25, 125 and 250 µg/mL) of the different solvent extracts. Three independent experiments were performed in triplicate. All values are mean ± SD, *n* = 3

The anticancer potential of the different solvent extracts was assessed against breast cancer (MDA-MB-231 cells) and myeloma (U266 and JJN3 cells) using the MTT assay, with incubation periods of 24–48 h. Intriguingly, the EtAC extract exhibited the highest potency, demonstrating significant activity against human myeloma cells with IC50 values of 62.12 ± 1.09 µg/mL and 43.39 ± 1.27 µg/mL after 24 and 48 h of incubation, respectively (Fig. 1c and d). In stark contrast, the MeOH extract showed comparatively reduced activity, resulting in a twofold decrease against U266 cells, with IC50 values of 131.6 ± 1.1 µg/mL and 117.3 ± 1.06 µg/mL for both timeframes (Fig. 1c and d). The Hex extract demonstrated the weakest effect on U266 cells, achieving a maximum inhibition of 30% even at the highest dose of 250 µg/mL (Fig. 1). In the case of MDA-MB-231 cells, a distinct pattern emerged. After 24 h of incubation (Fig. 1a), the maximum effect did not exceed 40% for all extracts, resulting in IC50 values of 217.2 ± 1.56 µg/mL, 358 ± 1.61 µg/mL, and 388.9 ± 1.56 µg/mL for EtAC, MeOH, and Hex extracts, respectively. Both the MeOH and EtAC extracts exhibited more potent activity against MDA-MB-231 cells after 48 h of treatment (Fig. 1b), with the MeOH extract revealing an IC50 of 112.7 ± 1.44 µg/mL, and the EtAC extract showing an IC50 of 131 ± 1.24 µg/mL. These findings highlight the divergent impact of the extracts on the different cell lines, with EtAC extract excelling against multiple myeloma cells and the MeOH extract proving effectiveness against MDA-MB-231 cells over the 48-h period.

### Cellular effects study

**Fig. 2:**
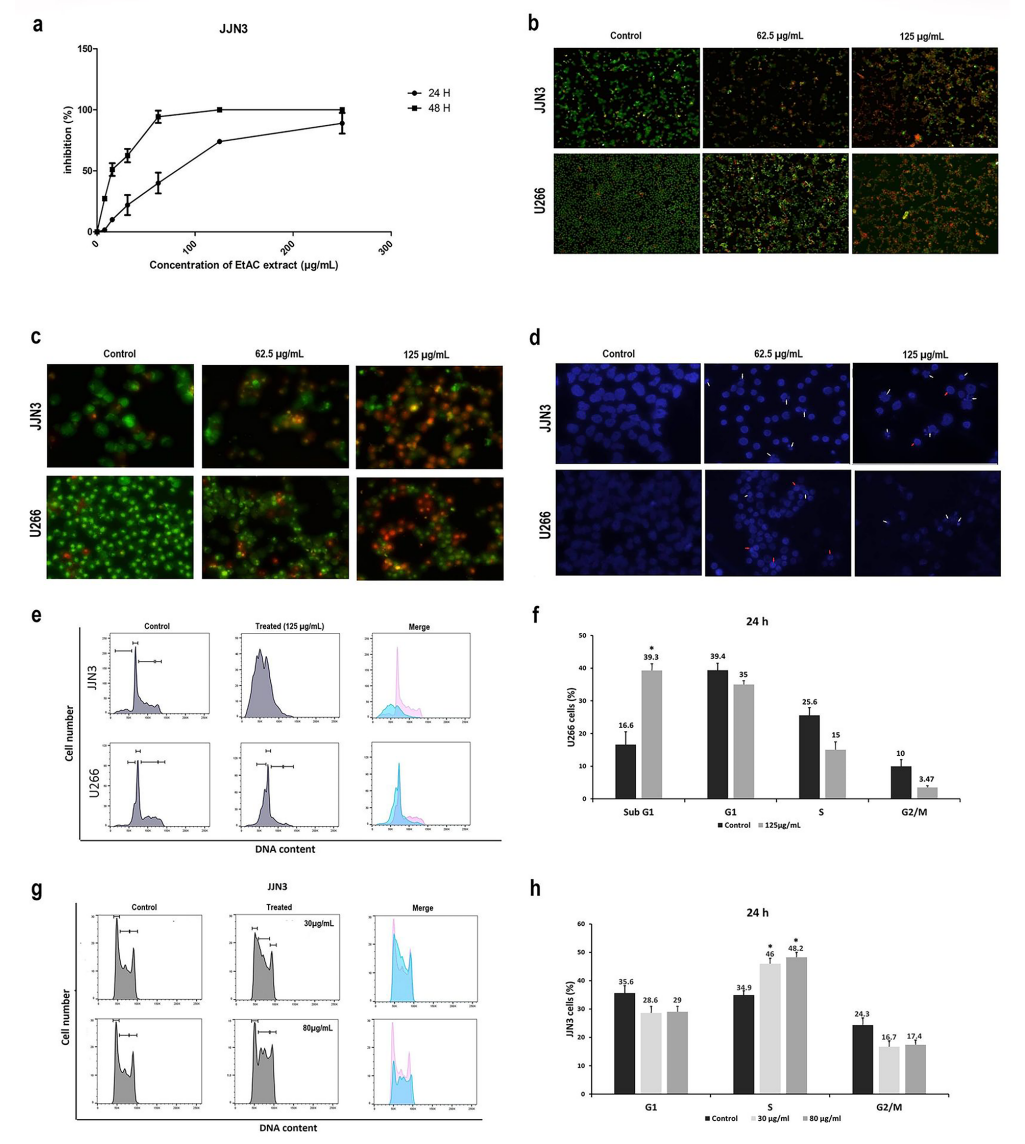
Evaluation of cytostatic and cytotoxic effects of EtAC extract of AC on myeloma cells. (**a**) The cytotoxic impact of the extract on JJN3 cells was assessed using the MTT assay. Cells were exposed to escalating concentrations (7.78, 15.56, 31.125, 62.25, 125, and 250 µg/mL) of EtAC solvent extract for 24–48 h. Reported values represent means ± SD from three independent experiments. Myeloma cells were treated with EtAC extract (62.25, 125 µg/mL) for 24 h. Subsequently, U266 and JJN3 cells were stained with acridine orange/PI, two fluorescent dyes, and examined under darkfield fluorescence microscopy, allowing distinct identification of viable, nonviable, and apoptotic cells. Images **b** and **c** depict representative magnifications of 100x and 400x, respectively. Image **d** illustrated nuclear changes in myeloma cells determined after DAPI staining and subsequent fluorescence microscopy analysis. Apoptotic cells are denoted by white arrows, while necrotic cells are marked by red arrows, with a magnification of x400. Cytometry profiles of cell cycle phases are displayed for JJN3 and U266 cells treated with 125 µg/mL EtAC extract (**e**) and JJN3 cells treated with 30 and 80 µg/mL EtAC extract **(g)**. Histograms were generated to visualize variations in different phases of the cell cycle in U266 **(f)** and JJN3 **(h)** cells. The graph presented the mean of three experiments, with error bars representing the standard deviation. P-values were calculated between treated cells and control, (*) denotes *p* < 0.05

The investigation aimed to elucidate the effects of the EtAC extract on human multiple myeloma cells, entailing a detailed analysis of its impact on cellular processes. Initially, we validated the cytotoxic activity of the EtAC extract on a second myeloma cell line called JJN3. Results demonstrated that the inhibitory effect of the EtAC extract on JJN3 cells is time-dependent, with IC50 values of 70.48 ± 1.08 µg/mL at 24 h and 14.62 ± 0.57 µg/mL at 48 h (Fig. 2a). Interestingly, JJN3 cells exhibited greater sensitivity to the EtAC extract compared to U266 cells after 48 h of treatment.

Subsequently, we aimed to discern the mechanisms by which the EtAC extract exerts its effects after 24 h of treatment. To achieve this, cell viability of U266 and JJN3 cells was assessed using acridine orange/PI double staining. Acridine orange penetrates both live and dead cells (green fluorescence), while PI penetrates only dead cells, emitting red fluorescence. Apoptotic cells can be marked by both acridine orange and PI, displaying color variation from yellow to orange. The results presented in Fig. 2b demonstrated predominant green staining in control cells (U266 and JJN3), in comparison to cells treated with a 62.5 µg/mL dose. There was a notable shift in staining from green to orange/red in JJN3 cells and a slight shift in U266 cells. Furthermore, an increase in the number of cells stained in red and orange was observed in both cell types compared to the control, particularly evident with an EtAC extract concentration of 125 µg/mL (Fig. 2b). Moreover, Fig. 2c vividly illustrates apoptotic cells (yellow or light orange), especially visible under high magnification (X400). These results suggest the induction of apoptotic and necrotic cell death by the EtAC extract in U266 and JJN3 cells.

Additionally, DAPI staining was used to visualize the morphological changes in the nuclei of U266 and JJN3 cell induced by 62.5 and 125 µg/mL of EtAC extract treatments. CytoVision analysis of images revealed stronger blue fluorescence intensity in the treated cells compared to the control (Fig. 2d). Moreover, a reduction in cell numbers was observed with increasing extract concentration, accompanied by a decrease in cell size. At an extract concentration of 62.5 µg/mL, microscopic observations revealed notable cellular transformations characterized by condensed or fragmented nuclei, indicative of apoptotic bodies. Additionally, in U266 cells, dispersed nuclei, typically associated with cells undergoing necrosis, were also observed.

Finally, the investigation of the EtAC extract’s impact on U266 and JJN3 cell cycle progression revealed considerable disruption in the cell cycle, particularly in the JJN3 cell line where a typical cell cycle profile was no longer observed (as presented in Fig. 2e). For the U266 cell line, cytometry profiles indicated the emergence of a cell population in the sub-G1 phase, representative of apoptotic cells. Additionally, FlowJo data analysis (Fig. 2f) indicated a significant increase in the percentage of cells in the sub-G1 phase in U266 cells compared to control (39.3% vs. 16.6%). Subsequently, we opted to test lower concentrations (30 and 80 µg/mL) on the JJN3 cell line (Fig. 2g). At the concentration of 30 µg/mL of the EtAC extract, the results exhibited a blockade in the S phase with a 11% increase compared to the control (46% vs. 34.9%, *p* < 0.01). Similarly, this blockade was maintained with a concentration of 80 µg/mL (47.2% vs. 34.9%) as showed in Fig. 2h.

## Discussion

Plant-based research has become a captivating avenue in the quest for novel bioactive molecules with diverse therapeutic potential [13, 35, 36]. *Artemisia* has been a focal point of study due to its historical significance in traditional medicine and its potential in modern pharmacology. This genus is renowned for harboring a rich array of bioactive compounds, making it a valuable resource for extracting and isolating molecules with pharmaceutical promise [37]. In this context, AC, a member of the *Artemisia* genus, has garnered substantial interest due to its multifaceted properties [18]. In our study, we focus on analyzing the composition of AC and evaluating successive extraction method used to access its bioactive constituents. The success of extraction depends on the specific conditions used [38]. The choice of solvent significantly influences the extraction of molecules based on their polarity [39]. We scrutinize the outcomes of using MeOH, EtAC, and Hex as solvents, revealing that MeOH and EtAC extracts yield higher concentrations, whereas Hex yields lower amounts.

Phytosterols, resembling cholesterol and known for potential health benefits in disease prevention [40, 41], were quantified in our study. Stigmasterol and β-sitosterol, prominent phytosterols, were focused on. The Hex extract exhibited the highest phytosterol content (67.55 mg/g DE), indicating Hex’s effectiveness in extracting these nonpolar molecules from AC. In contrast, the EtAC extract displayed a moderate phytosterol content, suggesting its lesser effectiveness for phytosterols despite extracting some non-polar compounds. MeOH, a polar solvent, exhibited the lowest phytosterol content, suggesting its unsuitability for extracting these nonpolar compounds. This paper marks the initial documentation of the presence of stigmasterol and β-sitosterol in our Tunisian AC, specifically extracted using Hex and EtAC.

In our study, MeOH extract displayed a high concentration of polyphenols, including flavonoids and phenolic acids, with respective concentrations of 783.46 mg/g DE and 46.07 mg/g DE (refer to Tables 2 and 3). Compounds like 3,4-dicaffeoylquinic and 4,5-dicaffeoylquinic acids, found in significant amounts in the MeOH fraction, hold considerable pharmacological, nutraceutical, and cosmetic potential [38]. Conversely, Hex was less effective in extracting polar polyphenols. In contrast, EtAC exhibited proficiency in extracting polyphenols, albeit with a slightly different profile than MeOH. LC-MS analysis demonstrated that EtAC extract contained a substantial concentration of polyphenols, particularly flavonoids (773.68 mg/g DE). Compounds like cirsiliol and luteolin found in this extract are recognized for their potent antioxidant, anti-inflammatory, and anticancer activities [42, 43]. For instance, Bakchiche’s study reported phenolic (212.87 GAE/g) and flavonoid (75.96 RE/g) contents in Algerian AC MeOH extract [44, 45]. Additionally, the HPLC-DAD data from Belgacem and colleagues revealed that the n-butanol fraction of the methanol leaf extract of Tunisian AC exhibited higher phenolic content and antioxidant potential compared to the total leaf methanol extract. This fraction contained significant quantities of rutin and chlorogenic acid. In contrast, our study found that these compounds were either absent or present in trace amounts [46]. The fluctuations in polyphenol levels can be attributed to various factors, including geographical conditions, extract type, and especially the extraction method itself [39, 47]. Importantly, our work achieved the highest extraction rates of total polyphenols from the MeOH and EtAC extracts. In this case, Metoui’s study on Tunisian AC indicated that the maceration method was the best one for the extraction of total polyphenols (345.8 mg GAE/g dry extract) and total flavonoids (296.3 mg QE/g dry extract) when butanol and ethyl EtAC were used as solvents, respectively. They have also shown that the EtAC extract obtained with maceration method was the richest in phenolic compounds, such as luteolin (9874.9 µg/g DE) while the EtAC extract obtained by the fractionation method showed the highest antioxidant (DPPH method) activity with an IC_50_ value of 11.0 mg/L [47]. Our study linked antioxidant activity to extract composition. MeOH extract showed the highest activity (89.05% at 50 µg/mL) due to rich polyphenols, notably flavonoids and phenolic acids (829.53 mg/g DE). EtAC extract had slightly lower activity (74.19% with 774.07 mg/g DE polyphenols) and Hex extract had the weakest activity (37.87% with 1.27 mg/g DE polyphenols). The findings underscore the robust antioxidant potential of AC extracts, as seen in previous studies revealing an 86% anti-radical activity in a 50 mg/mL aqueous AC extract from Tunisian research [48], and significant DPPH scavenging activity in MeOH and EtAC extracts with IC50 values of 6.0 and 10.0 µg/mL, respectively, as reported by Megdiche-Ksouri et al. [49].

In this study, we thoroughly analyzed, for the first time three specific terpenes-lupeol, oleanolic acid, and ursolic acid. EtAC extract displayed the highest terpene content, notably 34.03 mg/g DE of lupeol and 6.37 mg/g DE of oleanolic acid. Conversely, Hex extract showed notably lower terpene content compared to EtAC, lacking lupeol despite significant quantities of oleanolic acid and ursolic acid. Similarly, MeOH extract had the lowest terpene content, potentially due to the nature of these terpenes being less soluble in MeOH. Indeed, according to the literature, the identification of lupeol, oleanolic acid, and ursolic acid in the leaves of AC, particularly in the EtAC extract, represents a first in our study. However, other studies have reported the presence of two sesquiterpenes in the MeOH extracts of AC *subsp. Maritima* extracts [49], highlighting the crucial role of solvent selection in targeted terpene extraction, consistent with established practices [50]. Medicinal plants, owing to their terpene composition, are gaining attention as potential alternatives in combating multi-resistant bacterial diseases [51, 52]. Our study assessed AC extracts’ antibacterial and antifungal potential. Remarkably, the EtAC extract exhibited potent antibacterial activity against *Staphylococcus aureus* and *Pseudomonas aeruginosa*, with 12.22 mm and 7.66 mm inhibition zones at 25 µg/mL. In contrast, Hex, containing fewer terpenes, displayed milder antibacterial activity (approx. 6 mm), while the MeOH extract showed no antibacterial effect. Interestingly, in the case of the Polish AC subsp. *lednicensis*, the Hex leaf extract exhibited a weak effect on those strains, with a MIC exceeding 5000 mg/L [53]. Moreover, the lack of antibacterial activity in the MeOH extract could potentially be explained by the LC-MS analysis, which revealed the absence of certain flavonoids (quercetin, catechin, and apigenin) widely known for their antibacterial activity [54]. Furthermore, our results are consistent with the findings of the El Abed study, which demonstrated that the MeOH extract of leaves of Tunisian AC was not effective against *Pseudomonas aeruginosa* even at 10 mg/mL. Conversely, they showed a significant antibacterial effect of this extract against *Staphylococcus aureus*, with a 20 mm inhibition zone at the mentioned concentration [55].

Our results highlight, for the first time, the promising potential of AC-EtAC extract as an antibacterial source, particularly against the multi-resistant strain of *Pseudomonas aeruginosa*. Regarding the antifungal assessment against *Aspergillus flavus* and *Aspergillus niger*, all extracts demonstrated strong efficacy. At 50 µg/mL, both MeOH and EtAC extracts exhibited significant antifungal activity against *Aspergillus flavus*, with inhibition rates of 92.39% and 85.61%, respectively. Surprisingly, Hex displayed substantial inhibitory effects against both fungi, suggesting the potential contribution of components such as terpenes. This study explores, for the first time, the antifungal potential of AC against these species, enhancing the existing knowledge about the antimicrobial properties of the *Artemisia* genus.

The cytotoxicity evaluation of AC extracts against MDA-MB-231 and U266 cancer cell lines revealed varying inhibitory effects. MeOH and EtAC extracts displayed substantial cytotoxicity against both cell lines, whereas the Hex extract showed limited impact. Specifically, MeOH and EtAC extracts exhibited notable cytotoxicity against MDA-MB-231 cells after 48 h of treatment. Conversely, the EtAC extract demonstrated significant cytotoxicity against U266 multiple myeloma cells. These cytotoxic findings, conducted for the first time on AC, indicate a strong therapeutic potential of AC inhibiting cancer cell growth, especially against the incurable multiple myeloma cancer. However, there are limited literature demonstrating the efficacy of AC-derived compounds in targeting cancer cells. Specifically, Metoui’s study reported that the cirsiliol compound isolated from AC dried leaves exhibited cytotoxic activity against ovarian cell lines OVCAR-3 and IGROV-1, as well as the human colon cell line HCT-116, at 15 µM, with inhibition percentage respective values of 53.7, 48.8 and 40.9% [47, 56]. To enhance our knowledge about this AC property on this hematological cancer, we have chosen a second myeloma cell line named JJN3 to investigate the EtAC extract’s impact on cellular processes. The MTT assay revealed time-dependent inhibition in JJN3 cells, with a threefold higher sensitivity observed compared to U266 cells when the treatment duration was 48 h. Further analysis indicated that the extract induced apoptotic and necrotic cell death, as evidenced by acridine orange/PI staining and DAPI staining, demonstrating altered nuclear morphology, and confirming apoptotic bodies and necrotic cell features. Additionally, flow cytometry assessment demonstrated significant disruptions in the cell cycle of both cell lines upon exposure to the EtAC extract. Specifically, a notable increase in the percentage of cells in the sub-G1 phase was observed in U266 cells, while a blockade in the S phase was noted in JJN3 cells. The uniqueness of this research lies in its innovative approach, being the first to evaluate AC extracts in this manner against these specific cancer cell lines. Future studies can concentrate on isolating and characterizing specific bioactive compounds from AC extracts that interact with myeloma cancer cells. This approach can aid in identifying lead compounds with pharmaceutical potential and elucidating their modes of action, whether used alone or in combination with chemotherapy.

## Conclusions

The investigation into Tunisian AC extracts has highlighted their importance as a rich source of bioactive compounds with multifaceted therapeutic potential. The innovative approach of the study lies in comparing different solvent-based extraction methods and their influence on the observed biological effects. AC extracts demonstrated variations in the extraction of phytosterols, polyphenolic compounds, and terpenes. Significantly, this study represents the first documentation of specific compounds, such as lupeol and oleanolic acid, stigmasterol and β-sitosterol, found in Tunisian AC leaf extracts. Additionally, both the MeOH and EtAC extracts exhibited robust antioxidant and antifungal activities. Furthermore, this study represents a pioneering assessment of the EtAC extract, revealing its potential in multiple myeloma treatment by inducing apoptotic and necrotic cell death in resistant cells, unveiling previously undiscovered therapeutic significance.

### Electronic supplementary material

Below is the link to the electronic supplementary material.

Supplementary Material 1

## Data Availability

No datasets were generated or analysed during the current study.

## Abbreviations

AC: *Artemisia campestris* L
Hex: hexane
EtAC: ethyl acetate
MeOH: methanol
CFU: colony-forming units
DMSO: dimethyl sulfoxide
DPPH: (2,2-diphenyl-1-picrylhydrazyl)
MTT: 3-(4,5-dimethylthiazol-2-yl)-2,5-diphenyltetrazolium bromide
PI: propidium iodide
DAPI: 4’,6-diamidino-2-phénylindole
FBS: fetal bovine serum
PBS: Phosphate buffered-saline
DE: dry extract

## References

[1] Alam N, Wright AK, Ashcroft DM, Renehan AG (2020). Cancer and cardiovascular disease. Lancet.

[2] Ganesh K, Massagué J (2021). Targeting metastatic cancer. Nat Med.

[3] Dharshini LCP, Rasmi RR, Kathirvelan C, Kumar KM, Saradhadevi KM, Sakthivel KM (2023). Regulatory Components of Oxidative Stress and Inflammation and their Complex Interplay in Carcinogenesis. Appl Biochem Biotechnol.

[4] Limam I, Abdelkarim M, El Ayeb M, Crepin M, Marrakchi N, Di Benedetto M (2023). Disintegrin-like protein strategy to Inhibit Aggressive Triple-negative breast Cancer. Int J Mol Sci.

[5] Heider M, Nickel K, Högner M, Bassermann F (2021). Multiple myeloma: Molecular Pathogenesis and Disease Evolution. Oncol Res Treat.

[6] Bray F, Ferlay J, Soerjomataram I, Siegel RL, Torre LA, Jemal A (2018). Global cancer statistics 2018: GLOBOCAN estimates of incidence and mortality worldwide for 36 cancers in 185 countries. CA Cancer J Clin.

[7] Lamorte D, Faraone I, Laurenzana I, Milella L, Trino S, De Luca L (2018). Future in the past: Azorella Glabra Wedd. As a source of New Natural compounds with Antiproliferative and cytotoxic activity on multiple myeloma cells. Int J Mol Sci.

[8] Yusuf K, Sampath V, Umar S (2023). Bacterial infections and Cancer: Exploring this Association and its implications for Cancer patients. Int J Mol Sci.

[9] Harvey AL, Edrada-Ebel R, Quinn RJ (2015). The re-emergence of natural products for drug discovery in the genomics era. Nat Rev Drug Discov.

[10] Zhang W-J, Wang S, Kang C, Lv C, Zhou L, Huang L-Q (2020). Pharmacodynamic material basis of traditional Chinese medicine based on biomacromolecules: a review. Plant Methods.

[11] Jaradat N, Abualhasan M, Hawash M, Qadi M, Al-Maharik N, Abdallah S (2023). Chromatography analysis, in light of vitro antioxidant, antidiabetic, antiobesity, anti-inflammatory, antimicrobial, anticancer, and three-dimensional cancer spheroids’ formation blocking activities of Laurus nobilis aromatic oil from Palestine. Chem Biol Technol Agric.

[12] Limam I, Abdelkarim M, Essid R, Chahbi A, Fathallah M, Elkahoui S (2020). Olea europaea L. Cv. Chetoui leaf and stem hydromethanolic extracts suppress proliferation and promote apoptosis via caspase signaling on human multiple myeloma cells. Eur J Integr Med.

[13] Limam I, Ben Aissa-Fennira F, Essid R, Chahbi A, Kefi S, Mkadmini K (2021). Hydromethanolic root and aerial part extracts from Echium Arenarium Guss suppress proliferation and induce apoptosis of multiple myeloma cells through mitochondrial pathway. Environ Toxicol.

[14] Dehelean CA, Marcovici I, Soica C, Mioc M, Coricovac D, Iurciuc S (2021). Plant-Derived Anticancer compounds as New perspectives in Drug Discovery and Alternative Therapy. Molecules.

[15] Essghaier B, Toukabri N, Dridi R, Hannachi H, Limam I, Mottola F (2022). First Report of the biosynthesis and characterization of silver nanoparticles using Scabiosa atropurpurea subsp. maritima Fruit extracts and their antioxidant, Antimicrobial and Cytotoxic properties. Nanomater Basel Switz.

[16] Oueslati S, Gharsalli W, Abdelkarim M, Ben Aissa-Fennira F, Ksouri R (2018). Biochemical evaluation and exploration of the antioxidant, antibacterial and anticancer potential of Zingiber officinale. J New Sci.

[17] Hussain A, Hayat MQ, Bokhari SAI (2021). Artemisia chamaemelifolia Vill: a rare species of genus Artemisia (Asteraceae-Anthemideae) now present in the northeast (Gilgit-Baltistan) region of Pakistan. Biol (Bratisl).

[18] Dib I, El Alaoui-Faris FE (2019). Artemisia campestris L.: review on taxonomical aspects, cytogeography, biological activities and bioactive compounds. Biomed Pharmacother.

[19] Hendel N, Djamel S, Madani̇ S, Selloum M, Boussakra F, Driche O (2021). Screening for in vitro antioxidant activity and antifungal effect of Artemisia campestris L. Int J Agric Environ Food Sci.

[20] Dib I, Angenot L, Mihamou A, Ziyyat A, Tits M (2017). Artemisia campestris L.: Ethnomedicinal, phytochemical and pharmacological review. J Herb Med.

[21] Jabri M-A, Tounsi H, Abdellaoui A, Marzouki L, Sebai H (2018). Protective effects of Artemisia campestris extract against gastric acid reflux-induced esophageal mucosa injuries. Pathophysiol off J Int Soc Pathophysiol.

[22] Abubakar AR, Haque M (2020). Preparation of Medicinal plants: basic extraction and fractionation procedures for experimental purposes. J Pharm Bioallied Sci.

[23] Cheraif K, Bakchiche B, Gherib A, Bardaweel SK, Çol Ayvaz M, Flamini G (2020). Chemical composition, Antioxidant, Anti-Tyrosinase, Anti-cholinesterase and cytotoxic activities of essential oils of six Algerian plants. Mol Basel Switz.

[24] Rafińska K, Pomastowski P, Rudnicka J, Krakowska A, Maruśka A, Narkute M (2019). Effect of solvent and extraction technique on composition and biological activity of Lepidium sativum extracts. Food Chem.

[25] Cuénod A, Pottier-Alapetite G, Labbé A. Flore analytique et synoptique de la Tunisie: Cryptogames vasculaires gymnospermes et monocotylédones [Internet]. Office de l’expérimentation et de la vulgarisation agricoles de Tunisie. Tunisie; 1954. https://www.bibliotheque.nat.tn/BNTK/doc/SYRACUSE/858394/flore-analytique-et-synoptique-de-la-tunisie?_lg=fr-FR.

[26] Le Floc’h E, Boulos L, Véla E (2010). Catalogue synonymique commenté de la flore de tunisie. [2è édition].

[27] Pottier-Alapetite G. Flore de Tunisie. Angiospermes-dicotylédones. Gamopétales, première partie [Internet]. Ministère de l’Enseignement Supérieur et de la Recherche Scientifique et le Ministère de l’Agriculture. Tunisie; 1981. https://bibdigital.rjb.csic.es/records/item/12768-redirection.

[28] Jaouadi O, Limam I, Abdelkarim M, Berred E, Chahbi A, Caillot M (2021). 5,6-Epoxycholesterol isomers induce Oxiapoptophagy in Myeloma cells. Cancers.

[29] Azmir J, Zaidul ISM, Rahman MM, Sharif KM, Mohamed A, Sahena F (2013). Techniques for extraction of bioactive compounds from plant materials: a review. J Food Eng.

[30] López-Fernández O, Domínguez R, Pateiro M, Munekata PES, Rocchetti G, Lorenzo JM (2020). Determination of Polyphenols using Liquid Chromatography-Tandem Mass Spectrometry technique (LC-MS/MS): a review. Antioxid Basel Switz.

[31] Kirby AJ, Schmidt RJ (1997). The antioxidant activity of Chinese herbs for eczema and of placebo herbs — I. J Ethnopharmacol.

[32] Celiktas OY, Kocabas EEH, Bedir E, Sukan FV, Ozek T, Baser KHC (2007). Antimicrobial activities of methanol extracts and essential oils of Rosmarinus officinalis, depending on location and seasonal variations. Food Chem.

[33] Bendimerad N, Taleb Bendiab SA, Benabadji AB, Fernandez X, Valette L, Lizzani-Cuvelier L (2005). Composition and antibacterial activity of Pseudocytisus Integrifolius (Salisb.) Essential oil from Algeria. J Agric Food Chem.

[34] Abdelkarim M, Younes KB, Limam I, Guermazi R, ElGaaied ABA, Aissa-Fennira FB. 3,6-dichloro-1,2,4,5-Tetrazine assayed at high doses in the metastatic breast Cancer cell line MDA-MB-231 reduces cell numbers and induces apoptosis. Curr Bioact Compd. 16:546–50.

[35] Poojary MM, Vishnumurthy KA, Vasudeva Adhikari A (2015). Extraction, characterization and biological studies of phytochemicals from Mammea suriga. J Pharm Anal.

[36] Bhatt SC, Naik B, Kumar V, Gupta AK, Kumar S, Preet MS (2023). Untapped potential of non-conventional rubus species: bioactivity, nutrition, and livelihood opportunities. Plant Methods.

[37] Kordali S, Cakir A, Mavi A, Kilic H, Yildirim A (2005). Screening of Chemical Composition and antifungal and antioxidant activities of the essential oils from three Turkish Artemisia species. J Agric Food Chem.

[38] Alara OR, Abdurahman NH, Ukaegbu CI (2021). Extraction of phenolic compounds: a review. Curr Res Food Sci.

[39] Hayat J, Akodad M, Moumen A, Baghour M, Skalli A, Ezrari S (2020). Phytochemical screening, polyphenols, flavonoids and tannin content, antioxidant activities and FTIR characterization of Marrubium vulgare L. from 2 different localities of Northeast of Morocco. Heliyon.

[40] Cristoni A, Di Pierro F, Bombardelli E (2000). Botanical derivatives for the prostate. Fitoterapia.

[41] Ms U, Ferdosh S, Haque Akanda MJ, Ghafoor K, R AH, Ali ME (2018). Techniques for the extraction of phytosterols and their benefits in human health: a review. Sep Sci Technol.

[42] Carlini L, Tancreda G, Iobbi V, Caicci F, Bruno S, Esposito A (2022). The Flavone Cirsiliol from Salvia x Jamensis binds the F1 moiety of ATP synthase, modulating free radical production. Cells.

[43] Kang KA, Piao MJ, Ryu YS, Hyun YJ, Park JE, Shilnikova K (2017). Luteolin induces apoptotic cell death via antioxidant activity in human colon cancer cells. Int J Oncol.

[44] Akrout A, Gonzalez LA, El Jani H, Madrid PC (2011). Antioxidant and antitumor activities of Artemisia campestris and Thymelaea hirsuta from southern Tunisia. Food Chem Toxicol Int J Publ Br Ind Biol Res Assoc.

[45] Bakchiche B, GÖren AC, AydoĞmuŞ Z, Mataracikara E, Ghareeb MA (2022). Artemisia campestris and artemisia herbaalba: lc-hresi-ms profile alongside their antioxidant and antimicrobial evaluation. ACTA Pharm Sci.

[46] Belgacem A, Senejoux F, Felgines C, Fraisse D, Bitri L, Khemiri I (2022). Anti-obesity effects of the n-butanol fraction of the methanolic leaf extract of Artemisia campestris from Tunisian pharmacopeia in male Wistar rats. J Complement Integr Med.

[47] Metoui R, Mighri H, Bouajila J, Znati M, El-Jani H, Akrout A (2022). Artemisia campestris dried leaf extracts: effects of different extraction methods and solvents on phenolic composition and biological activities. South Afr J Bot.

[48] Ghlissi Z, Sayari N, Kallel R, Bougatef A, Sahnoun Z (2016). Antioxidant, antibacterial, anti-inflammatory and wound healing effects of Artemisia campestris aqueous extract in rat. Biomed Pharmacother.

[49] Megdiche-Ksouri W, Trabelsi N, Mkadmini K, Bourgou S, Noumi A, Snoussi M (2015). Artemisia campestris phenolic compounds have antioxidant and antimicrobial activity. Ind Crops Prod.

[50] Jiang Z, Kempinski C, Chappell J (2016). Extraction and analysis of Terpenes/Terpenoids. Curr Protoc Plant Biol.

[51] Siddique HR, Saleem M (2011). Beneficial health effects of lupeol triterpene: a review of preclinical studies. Life Sci.

[52] Perveen S, Alqahtani J, Orfali R, Aati HY, Al-Taweel AM, Ibrahim TA (2020). Antibacterial and antifungal sesquiterpenoids from Aerial Parts of Anvillea Garcinii. Molecules.

[53] Trifan A, Czerwińska ME, Mardari C, Zengin G, Sinan KI, Korona-Glowniak I (2022). Exploring the Artemisia Genus: an insight into the phytochemical and multi-biological potential of A. Campestris subsp. lednicensis (Spreng.) Greuter & Raab-Straube. Plants.

[54] Veiko AG, Olchowik-Grabarek E, Sekowski S, Roszkowska A, Lapshina EA, Dobrzynska I (2023). Antimicrobial activity of Quercetin, Naringenin and Catechin: flavonoids inhibit Staphylococcus aureus-Induced Hemolysis and modify membranes of Bacteria and erythrocytes. Molecules.

[55] El Abed N, Fatma G, Mejri M, Marzouki MN, Ben Hadj Ahmed S, PHYTOCHEMICAL SCREENING AND ASSESSMENT OF ANTIOXIDANT, ANTIBACTERIAL AND CYTOTOXICITY ACTIVITIES OF FIVE TUNISIAN MEDICINAL PLANTS How to Cite This Article (2014). Int J Pharm Res BIO-Sci.

[56] Metoui R, Bouajila J, Znati M, Cazaux S, Neffati M, Akrout A (2017). Bioactive flavones isolated from Tunisian Artemisia campestris L. leaves. Cell Mol Biol.

