# Supplementary material for: Tunisian Artemisia campestris L.: a potential therapeutic agent against myeloma - phytochemical and pharmacological insights
Source: Plant Methods. 2024 May 2;20:59. doi: 10.1186/s13007-024-01185-4 (PMC11067135; doi:10.1186/s13007-024-01185-4)
Supplement: Supplementary file 1 — Supplementary Material 1 [file 13007_2024_1185_MOESM1_ESM.docx]

**Table S1: LC-MS Analytical method parameters**

| **Compounds** | **m/z** | **Retention time (min)** |
| --- | --- | --- |
| **Phenolic Acids** |  |  |
| Caffeic acid | 179 | 14.499 |
| Chlorogenic acid | 353.00 | 9.092 |
| Frulic acid | 193 | 23.100 |
| Gallic Acid | 169.00 | 3.988 |
| Quinic acid | 191 | 2.041 |
| Protocatechuic acid | 153.00 | 6.907 |
| Rosmarinic acid | 359 | 26.450 |
| Salviolinic acid | 717 | 28.121 |
| Syringic acid | 197.00 | 16.078 |
| Trans-Cinnamic acid | 147.00 | 32.053 |
| 1,3-dicaffeoylquinic acid | 515.00 | 16.933 |
| 3,4-dicaffeoylquinic acid | 515.00 | 24.982 |
| 4,5-di-O-caffeoylquinic acid | 515.00 | 26.827 |
|  |  |  |
| **Flavonoids** |  |  |
| Acacetin | 283 | 40.253 |
| Apigenin | 269.00 | 34.589 |
| Apigenin-7-O-glucoside | 431.00 | 26.930 |
| Catechin | 289 | 11.184 |
| Cirsilineol | 343.00 | 38.619 |
| Cirsiliol | 329.00 | 35.296 |
| Epicatechin | 289 | 16.529 |
| Luteolin | 285 | 35.126 |
| Luteolin-7-O-glucoside | 447.00 | 24.626 |
| Quercetin | 447.00 | 26.827 |
| Rutin | 609 | 24.122 |
| Silymarin | 481.00 | 33.807 |
|  |  |  |
| **Terpenes** |  |  |
| Lupeol | 409.00 | 16.506 |
| Oleanolic acid | 439.00 | 16.306 |
| Ursolic acid | 439.00 | 16.738 |
|  |  |  |
| **Phytosterols** |  |  |
| Stigmasterol | 13.900 | 395.30 |
| β-sitosterol | 15.621 | 397.40 |

The control of the LC and MS hardware. as well as the chromatographic and spectral data processing. was carried out through the Shimadzu LabSolutions LC-MS software. Chromatographic peaks were identified by evaluating their retention times at their specific m/z ratio compared to that of analytical standards. and analyte quantification was performed using external calibration curves.
